# Molecular Evolutionary Rate Predicts Intraspecific Genetic Polymorphism and Species-Specific Selection

**DOI:** 10.3390/genes13040708

**Published:** 2022-04-17

**Authors:** Jiaqi Wu, Takahiro Yonezawa, Hirohisa Kishino

**Affiliations:** 1 Department of Molecular Life Science, Tokai University School of Medicine, Isehara 259-1193, Japan; 2 Faculty of Agriculture, Tokyo University of Agriculture, Atsugi 243-0034, Japan; cyclotis@gmail.com; 3 Graduate School of Agricultural and Life Sciences, The University of Tokyo, Bunkyo Ward, Tokyo 113-8657, Japan; 4 The Research Institute of Evolutionary Biology, Tokyo 138-0098, Japan; 5 AI/Data Science Social Implementation Laboratory, Chuo University, Tokyo 112-8551, Japan

**Keywords:** long-term molecular evolutionary rates, genetic diversity, human-specific evolution, species-specific evolution, gene-specific molecular evolutionary rates, gene effect, locus effect

## Abstract

It is unknown what determines genetic diversity and how genetic diversity is associated with various biological traits. In this work, we provide insight into these issues. By comparing genetic variation of 14,671 mammalian gene trees with thousands of individual human, chimpanzee, gorilla, mouse, and dog/wolf genomes, we found that intraspecific genetic diversity can be predicted by long-term molecular evolutionary rates rather than de novo mutation rates. This relationship was established during the early stage of mammalian evolution. Moreover, we developed a method to detect fluctuations of species-specific selection on genes based on the deviations of intraspecific genetic diversity predicted from long-term rates. We showed that the evolution of epithelial cells, rather than connective tissue, mainly contributed to morphological evolution of different species. For humans, evolution of the immune system and selective sweeps caused by infectious diseases are the most representative examples of adaptive evolution.

## 1. Introduction

What determines genetic diversity and how genetic polymorphism is shaped within species are core questions in evolutionary biology. It is generally accepted that replication errors in genomes of germline cells, known as de novo mutations, are the source of genetic diversity, and that demographic history (population history and effective population size) and selection shape genetic polymorphisms within species [1,2,3]. In particular background selection, which describes reduction in genetic diversity in non-deleterious loci caused by selection against linked deleterious mutations, largely explains the patterns of genome-wide distribution of polymorphic sites [1,4,5]. Furthermore, a longstanding enigmatic issue known as Lewontin’s paradox has also been mostly resolved by our enhanced understanding of background selection in the last decade [6]. This paradox tells us that the distribution of nucleotide diversity, which is generally considered to be determined by the effective population size (Ne), and variation in actual population size differ by many orders of magnitude [7]. When a gene variant, or an allele, is fixed in the species, it becomes a substitution and contributes to interspecific genetic diversity. However, the connections between genetic polymorphisms within species and species-level substitutions are still controversial issue.

As pointed out by R. A. Fisher, the majority of mutations in a population are lost by chance [8]. According to Motoo Kimura’s neutral theory of molecular evolution, most mutations are either deleterious or selectively neutral [9,10]. Therefore, the molecular evolutionary rate of a gene is the product of the mutation rate and the proportion of neutral mutations [11]. In contrast, intraspecific genetic diversity is generated by mutations occurring along genealogies in a population that have neither reached fixation nor been lost. The shape of a genealogy depends on demographic history, and the lifespan of a mutation also depends on the selection intensity [12,13]. Here, we note that all genes in a genome share the same demographic history. Among-gene variation in genetic diversity thus largely reflects among-gene variation in the proportion of neutral mutations, as lineages originating from deleterious mutations are too short-lived to substantially contribute to present genetic diversity. Because among-gene variation in molecular evolutionary rates is also determined by the among-gene variation in a proportion of neutral mutations [14], we hypothesized that molecular evolutionary rates affect intraspecific genetic diversity.

If our hypothesis is correct, the genetic diversity within species can be predicted from their molecular evolutionary rates. Natural selection affects both intraspecific genetic diversity and molecular evolutionary rate, and the strength of natural selection is the main factor that shapes intraspecific genetic polymorphisms. Furthermore, testing this hypothesis can help to elucidate the role of background selection. Pouyet et al., 2018, measured average derived allele frequency per individual as a proxy for background selection, and demonstrated that it impacts up to 80–85% of the variants in the human genome [15]. Background selection is currently modeled based on the exon densities and genetic linkages. Therefore, if the strength of natural selection on the exon regions is taken into account to model the background selection, it will help to clarify intraspecific genetic polymorphisms and interspecific substitutions.

In addition, based on this idea, a novel method to infer genomic evolution could help detect signals of species-specific evolution. In this framework, the residues of the observed and predicted genetic diversities based on molecular evolutionary rates can be regarded as the signals of species-specific selection. Ever since Darwin, species-specific evolution has been considered a key factor in the diversification of living organisms and their remarkable adaptations to their environments. Nevertheless, the genetic background of species-specific evolution, and especially how selection pressures fluctuate along the history of a species, is still unclear, even in this genomic era. This difficulty largely stems from our limited knowledge about what determines genetic diversity and how genetic polymorphisms are shaped within species.

The aims of this study were to: (1) clarify how genetic polymorphisms have been shaped within species by testing our hypothesis that molecular evolutionary rates affect intraspecific genetic diversity based on phylogenomic analysis of mammals and population genomic analysis of five species (humans, chimpanzees, gorillas, mice, and dogs/wolves), and (2) develop a new method to infer genomic evolution that detects the signals of species-specific selection.

## 2. Materials and Methods

### 2.1. Measurement of Genetic Polymorphisms and De Novo Mutations

To test the above hypothesis that molecular evolutionary rates affect intraspecific genetic diversity, we analyzed and compared the genetic diversity of humans with the mammalian molecular evolutionary rates of each gene and focused on protein-coding regions. The proportion of segregating sites (q) is the most direct estimate of human intraspecific genetic diversity. Nucleotide diversity (π), the mean pairwise distance among sequences, more heavily weights older mutations. Singletons are rare variants that are only observed once in a sample, and can be considered as the collection of recent mutations. The allele frequency of singletons in autosomes is 1/5008, which is less than 0.02% of the number of sites in 2504 humans included in the 1000 Genomes Project data [16,17,18]. De novo mutations are obtained by comparing genomes of direct offspring with those of their parents, with the differences uncovered being the closest reflection of germ cell mutations [19]. By comparing the effects of molecular evolutionary rates on these different indices, we may gain some sense of the longevity of deleterious or slightly deleterious mutations that have been selected against [10].

In each population, natural selection works on phenotypes and on the gene sets that control them. Depending on the surrounding environment, the targets of natural selection may vary among species. To identify species-specific targets, we searched for significant deviations from the genetic diversity predicted by molecular evolutionary rates. Genes with unexpectedly high genetic diversity may be under reduced functional constraints, whereas those with unexpectedly low genetic diversity may be subject to enhanced constraints.

### 2.2. Probability of Intraspecific Polymorphism and Measurement of Molecular Evolutionary Rates

Genetic polymorphism within a species is generated by past mutations that have neither reached fixation nor been deleted. The probability *q* that a site is polymorphic is formulated as:

(1) $$q=\int_{t=0}^{\infty }dt\int_{-\infty }^{0}g\left(s\right)ds\;Pr\left(T>t|s,N_{e}\left(u\right),0\leq u\leq t\right)N_{e}\left(t\right)\nu .$$

Here, ν is the mutation rate, *s* and g(s) are the selection intensity and its distribution, and $N_{e}\left(u\right)$ is the effective population size at time u before present. We note that the probability of a positive value for s can be assumed to be negligible. T, which is the survival time of a mutation before either being fixed or lost in the population, depends on the selection intensity and population history. Ignoring the contribution of slightly deleterious mutations for simplicity, we considered the scenario in which genetic polymorphism is largely generated by neutral mutations. In this scenario, Equation (1) becomes:

(2) q=νpF,

where

$$F=\int_{t=0}^{\infty }Pr\left(T>t|s=0,N_{e}\left(u\right),0\leq u\leq t\right)2N_{e}\left(t\right)dt.$$

The probability that a mutation is selectively neutral, p, expresses the extent of the independence of the mutation from functional constraints. Although p varies among genes, F does not. As a result, among-gene variation in the proportion of segregating sites reflects either the variation in mutation rates or the variation of functional constraints.

Interspecific molecular evolution, in contrast, is based on mutations that have become fixed in populations. Assuming the above scenario, the neutral theory of molecular evolution expresses the molecular evolutionary rate as:

(3) $$r=2N_{e}\left(t\right)\nu \times p\times \frac{1}{2N_{e}\left(t\right)}=\nu p.$$

Although intraspecific genetic variation is many orders of magnitude lower than that generated during the evolution of all mammals, Equations (2) and (3) predict that the among-gene variation in the proportion of segregating sites is correlated with the among-gene variation in the molecular evolutionary rate.

The molecular evolutionary rate can be measured from the variation in total branch lengths of gene trees, which expresses the number of substitutions across the whole tree. Additionally, mean branch lengths of gene trees should well reflect the variation in evolutionary rates among genes, even in the presence of missing values, unless the resultant sub-trees of the (unobserved) gene trees are seriously skewed. Gene trees sometimes include extremely long terminal branches, even after careful inspection to control the quality of the alignment. Because mean branch length is sensitive to extremely long outlier branches, we also calculated the median branch length as a robust estimate of among-gene variation in molecular evolutionary rates.

### 2.3. Neutral tTheory Extended to Multiple-Gene Molecular Evolution

The well-accepted neutral theory of molecular evolution asserts that selectively advantageous mutations are rare among those mutations leading to interspecific variation [9]. As a result, the rate of molecular evolution is the product of the mutation rate, ν, and the proportion of neutral mutations, p [11]. By extending this relationship to the rates of multiple-gene molecular evolution, we obtained the branch lengths of gene trees using the following formula [14]:

$$b_{\left(i\right)}^{k}=c\times {\bar{p}}_{i}\times \left(t_{k}\times \nu_{k}\right)\times {\bar{p}}_{ik}.$$

Here, ${\bar{p}}_{i}$ represents the relative variability of gene i (i=1,…,N), and $t_{k}$ and $\nu_{k}$ are the evolutionary time span and mutation rate along branch k (k=1,…,M). By applying a two-way ANOVA-type Poisson regression to the scaled branch lengths ${\tilde{N}}_{\left(i\right)}^{k}=b_{\left(i\right)}^{k}\times L_{i}$ (where $L_{i}$ is the length of gene i), namely,

$$E\left[{\tilde{N}}_{\left(i\right)}^{k}\right]=c\times L_{i}\times \alpha_{i}\times \beta_{k}$$

$$where\sum_{i=1}^{N}L_{i}\times \alpha_{i}=\sum_{k=1}^{M}\beta_{k}=1,$$

we can estimate ${\bar{p}}_{i}$ and $t_{k}\times \nu_{k}$ based on the gene effect ${\hat{\alpha }}_{i}$ and branch effect ${\hat{\beta }}_{k}$. The interaction ${\bar{p}}_{ik}$, which captures the among-branch variation of functional constraints on a gene, is estimated as the ratio of ${\tilde{N}}_{\left(i\right)}^{k}$ to the predicted value. When genomes include a complete set of orthologous genes for all species, the maximum likelihood estimators of the gene effect and the branch effect can be obtained as follows:

(4) $${\hat{\alpha }}_{i}=\frac{\sum_{k=1}^{M}b_{\left(i\right)}^{k}}{\sum_{i=1}^{N}M\sum_{k=1}^{M}b_{\left(i\right)}^{k}\times L_{i}}$$

$${\hat{\beta }}_{k}=\frac{\sum_{i=1}^{N}b_{\left(i\right)}^{k}\times L_{i}}{\sum_{i=1}^{N}M\sum_{k=1}^{M}b_{\left(i\right)}^{k}\times L_{i}}$$

### 2.4. Summary Statistics Describing Variation in Long-Term Evolutionary Rates among Genes in a Genome

In this study, we investigated the possibility that intraspecific population genetic diversity is affected by the between-species long-term molecular evolutionary rate. Specifically, we contrasted the rate of segregation in humans with the rate of molecular evolution in mammals. As can be seen in Equation (4), the among-gene variation in the molecular evolutionary rate can be measured from the total branch lengths of gene trees.

In actual practice, however, gene trees often include missing taxa, partly because of incomplete sequencing, but mainly because of gene loss over the course of mammalian evolution. As a result, the among-gene variation in total branch lengths reflects both the among-gene variation in molecular evolutionary rate and variation in taxon sampling. To reduce the effect of differential taxon sampling, we used mean branch lengths:

$${\bar{b}}_{\left(i\right)}∶=\frac{1}{M}\overset{M}{\underset{k=1}{\sum }}b_{\left(i\right)}^{k}.$$

The variation in mean branch length should well reflect the variation in evolutionary rates among genes, even in the presence of missing values, unless the resultant sub-trees of the (unobserved) complete gene trees are seriously skewed.

The generated gene trees sometimes included extremely long terminal branches. Long branches in a gene tree may be ascribed to three phenomena: (1) sequencing or annotation errors, (2) bias caused by the model used for tree inference, or (3) lineage-specific selection. Even after careful inspection to control the alignment quality, however, we could not exclude the possibility of incomplete modeling of molecular evolution or lineage-specific selection in some cases. Because mean branch length is sensitive to extremely long outlier branches, we also calculated the median branch length as a robust estimate of the among-gene variation in molecular evolutionary rates:

$$r_{\left(i\right)}\propto {\tilde{b}}_{\left(i\right)}∶=\frac{b_{\left(i\right)\left[\#b_{\left(i\right)}÷2\right]}+b_{\left(i\right)\left[\#b_{\left(i\right)}÷2+1\right]}}{2}.$$

Regarding protein evolution at the amino acid level, more than half of the branches of some extremely conserved genes were of zero length; this resulted in a median branch length of 0, which would be problematic in the succeeding regression analysis. We therefore removed the five longest branches from each gene tree and obtained the truncated mean branch length, ${\hat{\bar{b}}}_{\left(i\right)}$, as an estimate of ${\bar{r}}_{\left(i\right)}$. Both ${\hat{\bar{b}}}_{\left(i\right)}$ and ${\tilde{b}}_{\left(i\right)}$ were used in this study.

### 2.5. Measuring the Joint Effects of Background Selection by Long-Term Evolutionary Rates and Hitchhiking in Non-Coding Regions

The genetic diversity at a genomic locus is reduced by the effects of selection on the surrounding region in the chromosome that accommodates the target locus. The effect of a small window at the genomic location $x_{i}$ is measured as:

$$1-\frac{u\left(x_{i}\right)\Delta x\cdot sh}{2{\left(sh+R\left(x_{i}\right)\right)}^{2}}.$$

Here, $u\left(x_{i}\right)$ is the deleterious mutation rate at the window, Δx is length of the window, sh is selective effect, and $R\left(x_{i}\right)$ is the recombination fraction between the window and the target locus [20]. In our study, we assumed background mutation rate is the same across all loci, and analyzed the effects of the surrounding exons that experienced different levels of selection. The neutral variation, $\pi_{0},$ is reduced by the joint effects of background selection as:

$$\pi =\pi_{0}\overset{m}{\underset{i=1}{\prod }}\left[1-\frac{U\Delta x\cdot sh_{i}}{2{\left(sh_{i}+R\left(x_{i}\right)\right)}^{2}}\right].$$

where the product runs over the windows in the exons of the chromosome. We introduce a model that describes the selective effect in terms of the long-term molecular evolutionary rate:

$$sh_{i}=exp\left(\alpha +\beta r_{i}\right).$$

where α is the gene effect of the gene that spans the window. Thus, we obtained:

(5) $$\pi =\pi_{0}\overset{m}{\underset{i=1}{\prod }}\left[1-\frac{U\cdot \Delta x\cdot exp\left(\alpha +\beta r_{i}\right)}{2{\left(exp\left(\alpha +\beta r_{i}\right)+R\left(x_{i}\right)\right)}^{2}}\right]\;\approx \pi_{0}exp\overset{m}{\underset{i=1}{\sum }}\left[\frac{-U\cdot \Delta x\cdot exp\left(\alpha +\beta r_{i}\right)}{2{\left(exp\left(\alpha +\beta r_{i}\right)+R\left(x_{i}\right)\right)}^{2}}\right].$$

For the de novo mutation rate of the genome, U, we assumed 1 × 10^−8^ per position per haploid genome based on [21].

We calculated π based on 1000 bp windows from 1000 Genomes Project Phase 3 data using VCFtools (v 0.1.16) [22]. We defined the intergenic region as more than 100,000 bp away from start or stop codons. Based on it, we assigned the exon, 5′-UTR, 3′-UTR, and intron regions of each window. Recombination rate $R\left(x_{i}\right)$ was summarized based on [23]. We used the African Caribbean in Barbados population’s recombination map from the 1000 Genomes Project to obtain the local recombination rate surrounding each window. We fitted Equation (5) by the nls (nonlinear least squares) function in R (v4.0.5) [24] and estimated the three parameters $\pi_{0}$, α, and β for 5′-UTR, 3′-UTR, and intron regions for each chromosome.

### 2.6. Observed and Corrected Correlations between Intraspecific Genetic Diversity and Long-Term Molecular Evolutionary Rates

To examine the effects of long-term molecular evolutionary rates on intraspecific genetic diversity, we calculated the correlation between the rate of molecular evolution $B=\left\{b_{\left(1\right)},b_{\left(2\right)},\ldots ,b_{\left(i\right)},\ldots \right\}$ and the number of segregating sites $K=\left\{K_{\left(1\right)},K_{\left(2\right)},\ldots ,K_{\left(i\right)},\ldots \right\}$. Because ${\bar{b}}_{\left(i\right)}$ and $K_{\left(i\right)}$ were accompanied by the random noise of estimation and sampling, we corrected the observed correlation by taking into account the sampling variance of each ${\bar{b}}_{\left(i\right)}$ and $K_{\left(i\right)}$.

To investigate the effect of sampling variance, we denoted the observed values of B and K as $\hat{B}$ and $\hat{K}$, where $E_{s}\left[\hat{B}|B\right]=B$, $E_{s}\left[\hat{K}|K\right]=K$, and the subscript “S” represents the sampling mean and variance. In addition, we used the subscript “G” to represent the among-gene mean and variance. The expectation of the observed correlation between $\hat{B}$ and $\hat{K}$, $r_{G}\left(\hat{B},\hat{K}\right)$, is contrasted with $r_{G}\left(B,K\right)$ as follows:

$$E_{s}\left[r_{G}\left(\hat{B},\hat{K}\right)\right]\sim \frac{E_{s}\left[COV_{G}\left(\hat{B},\hat{K}\right)\right]}{\sqrt{E_{s}\left[V_{G}\left(\hat{B}\right)\right]E_{s}\left[V_{G}\left(\hat{K}\right)\right]}}$$

$$=\frac{r_{G}\left(B,K\right)}{\sqrt{\left(1+\frac{E_{G}\left[V_{S}\left[\hat{B}|B\right]\right]}{V_{G}\left(B\right)}\right)\left(1+\frac{E_{G}\left[V_{S}\left[\hat{K}|K\right]\right]}{V_{G}\left(K\right)}\right)}}.$$

By evaluating the sampling variances of $\hat{B}$ and $\hat{K}$, we obtained the bias-corrected correlation as follows:

$$r_{G}\left(\hat{B},\hat{K}\right)∶=r_{G}\left(\hat{B},\hat{K}\right)\times \sqrt{\left(1+\frac{E_{G}\left[{\hat{V}}_{S}\left[\hat{B}|B\right]\right]}{V_{G}\left(\hat{B}\right)}\right)\left(1+\frac{E_{G}\left[{\hat{V}}_{S}\left[\hat{K}|K\right]\right]}{V_{G}\left(\hat{K}\right)}\right)}.$$

### 2.7. Sampling Variance of Mean Branch Lengths

We evaluated the sampling variance of branch lengths with reference to the Poisson random variables for the numbers of nucleotide or amino acid substitutions. Given that the branch length of gene *i* of branch *k*, ${\hat{b}}_{\left(i\right)}^{k},$ is the expected number of substitutions per site, we assumed that the stochastic variance of ${\hat{b}}_{\left(i\right)}^{k}l_{\left(i\right)}$ is approximated by the variance of a Poisson random variable with mean $b_{\left(i\right)}^{k}l_{\left(i\right)}$, where $l_{\left(i\right)}$ is the sequence length of gene i:

$$\hat{V}\left({\hat{b}}_{\left(i\right)}^{k}l_{\left(i\right)}\right)={\hat{b}}_{\left(i\right)}^{k}l_{\left(i\right)}.$$

The variance of the mean branch lengths of gene *i* is therefore evaluated as:

$$\hat{V}\left[{\bar{\hat{b}}}_{\left(i\right)}\right]=\hat{V}\left[\frac{1}{M}\overset{M}{\underset{k=1}{\sum }}{\hat{b}}_{\left(i\right)}^{k}\right]=\frac{1}{M^{2}}\sum_{k=1}^{M}\hat{V}\left({\hat{b}}_{\left(i\right)}^{k}\right)=\frac{1}{M^{2}}\sum_{k=1}^{M}\frac{{\hat{b}}_{\left(i\right)}^{k}}{l_{\left(i\right)}}=\frac{1}{Ml_{\left(i\right)}}{\bar{b}}_{\left(i\right)}.$$

Bootstrap resampling of columns in the alignment allows estimation of the variance of median and mean branch lengths. As an alternative approach, we developed a simple simulation method to evaluate the stochastic variance due to the randomness of substitutions. Given a gene tree, we generated the number of substitutions for each branch from the Poisson distribution, with its mean set to the product of the branch length and the gene length. We obtained the sample of the mean (and median) branch length by dividing the mean (and median) of the generated number of substitutions by the gene length.

### 2.8. Stochastic Variance of Intraspecific Genetic Diversity

If one assumes that mutations in a population are neutral and that the population is at equilibrium, then the variance in the number of segregating sites, *K*, is:

$$V\left(K\right)=E\left(K\right)+{\left(E\left(K\right)\right)}^{2}\frac{b_{n}}{a_{n}{}^{2}},$$

where $a_{n}=\sum_{i=1}^{n-1}\frac{1}{i}$ and $b_{n}=\sum_{i=1}^{n-1}\frac{1}{i^{2}}$. Here, *n* is the number of chromosomes in a sample from the population [25]. The first term reflects the randomness of mutations, and the second term measures the stochasticity of the genealogy. When the population is not at equilibrium but has its own history, the variance becomes:

$$V\left(K\right)=E\left(K\right)+{\left(E\left(K\right)\right)}^{2}\frac{V\left(T_{c}\right)}{E{\left(T_{c}\right)}^{2}}.$$

Here, $E\left(T_{c}\right)$ and $V\left(T_{c}\right)$ are the mean and variance of waiting times of coalescence events in genealogies [26]. Unfortunately, detailed information on genealogies is not available in most cases. We note, however, that the above variance formula is consistent with the variance formula of a negative binomial distribution. We therefore applied negative binomial regression with a log link:

$$\log\left[E\left(K\right)\right]=\log\left(L\right)+\alpha +\beta \log\left(BL\right)+\gamma D_{X}$$

where $L=\left\{l_{\left(1\right)},l_{\left(2\right)},\ldots ,l_{\left(i\right)},\ldots \right\}$ is the length of genes and BL is the mean branch length. $D_{X}$ is a dummy variable for genes on the *X* chromosome that incorporates differences in effective population size between an autosome and the *X* chromosome. We carried out this regression using the function glm.nb in the R package MASS [27]. The variance of *K* can be obtained by:

$$V\left(K\right)=E\left(K\right)+\frac{{\left[E\left(K\right)\right]}^{2}}{\hat{\theta }},$$

where $\hat{\theta }$ is the shape parameter of the negative binomial distribution.

We also applied the model:

$$E\left(K\right)=L\times \left[\alpha +\beta \left(BL\right)\right]\times \exp\left(\gamma D_{X}\right).$$

Maximum likelihood inference was conducted by applying the function optim. A positive value of α indicates intraspecific survival of mutations destined for deletion from the population on a macroevolutionary time scale.

### 2.9. Species Specificity: The Significant Deviation of Trait-Associated Gene Sets

Intraspecific genetic diversity is affected by long-term molecular evolutionary rates. Species-specific enhanced/reduced functional constraints may be observed as residuals of the prediction of the above negative binomial regression. For each gene in a trait-associated gene set, we calculated the lower-tailed *p*-value of the observed number of segregating sites based on the predicted value and the estimated scale parameter. To obtain the significance of the gene set, we standardized the *p*-values by transforming them to the percentiles of their corresponding standard normal distribution. The *p*-value of the sample mean of these transformed values was obtained using a t-distribution as a reference. The *t*-values measure the deviation from the predicted genetic diversities of genes in gene sets. Positive *t*-values indicate the existence of reduced functional constraints or diversifying selection in a human lineage, whereas negative values indicate the presence of enhanced functional constraints or purifying selection. Given the false discovery rate (FDR) value, significant genes were selected by the Benjamini and Hochberg procedure [28]. We tested the significance of the enhanced/reduced functional constraints on the disease-associated gene sets, the DisGeNET database [29] by contrasting the average deviation with the t distribution. We analyzed the gene sets that included at least two genes. Consequently, we analyzed 14,267 disease-related gene sets. We interpreted the disease-associated genes based on the disease category annotations in DisGenNET (v6.0) [29] and the MalaCard database [30].

### 2.10. Commonality and Specificity among Five Species

To generalize our findings, we also analyzed the genetic diversity of chimpanzees, orangutans, mice, and dogs/wolves. To visualize the commonality and specificity between species, we applied principal component analysis (PCA) to the proportion of segregating sites of five species and the long-term rate using 5560 single-copy genes. To interpret the genes that comprise the principal components, we examined the functions of the top 20 genes for each of the three PCs. For enrichment search [31], we set FDR = 0.05 as the significance cut-off value. To identify the genes and disease-associated gene sets unique to the species, we applied correspondence analysis (CA) to the negative log-transformed *p*-values.

### 2.11. Multiple-Alignment of Mammalian Genes

We downloaded 96 complete mammalian genomes from GenBank (https://www.ncbi.nlm.nih.gov/genbank/) and used a custom Perl script to extract protein-coding sequences of each species. A gene pool containing 21,350 mammalian genes was generated based on NCBI genomic annotation. We generated a multi-sequence file of all genes in the gene pool, and kept one sequence per species for each gene. Including genes that are shared by more than 70 out of 96 mammal species and shared with the human 1000 Genomes Project annotation (hg19 human genome assembly), 14,671 genes were used in this study. Among these genes, 5560 were previously reported as single-copy genes in class Mammalia in [14]. Alignments at the codon level were performed in Prank v.170427 [32]. Sites with less than 70% coverage among all species and sequences with less than 30% coverage among gene loci in the alignment were removed.

### 2.12. Inference of Gene Trees

We generated a maximum likelihood tree for each gene using IQ-TREE (v1.6.12) [33], which automatically performed model selection and determined the best data partitions. The best evolutionary model for each gene was independently selected based on the Bayesian information criterion and used to infer the gene tree. All gene trees were calculated using 1000 bootstrap replicates.

### 2.13. Population Data

We downloaded single-nucleotide polymorphism (SNP) data for 2504 humans (*Homo sapiens*) from the 1000 Genomes Project Phase 3 data [16,17,18]. SNP data for 60 common chimpanzees (*Pan troglodytes*), 31 gorillas (*Gorilla gorilla*), 35 laboratory mice (*Mus musculus*), and 127 dogs and wolves (*Canis lupus*) were collected from published papers and databases [34,35,36,37]. Information on de novo mutations in the human genome was collected from [19]. Human ancestral alleles were collected from [38]. Individual-derived alleles of each gene were collected by comparing SNPs of each individual with human ancestral sequences.

## 3. Results

### 3.1. The Effect of Long-Term Evolutionary Rates and Recombination on Shaping Genetic Diversity

**Figure 1:**
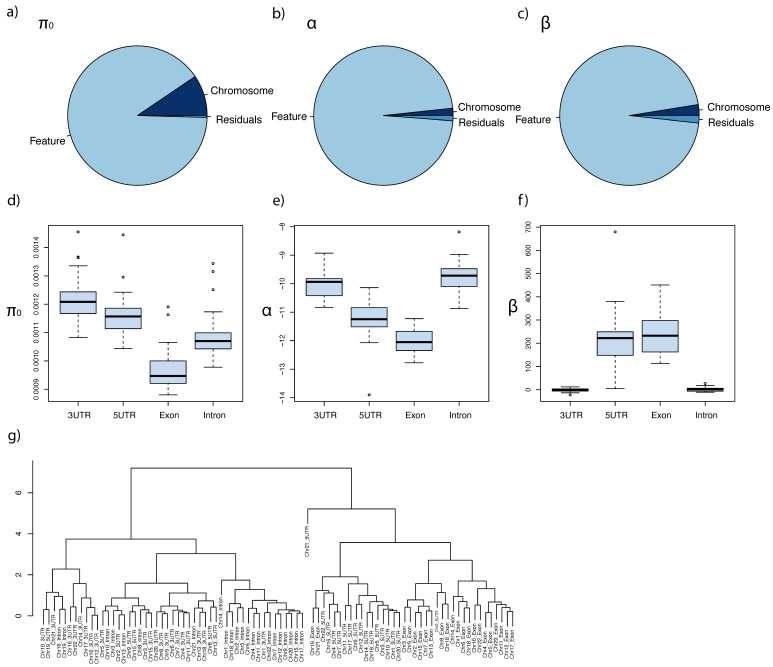
**Effect of long-term evolutionary rates and recombination on shaping genetic diversity.** π_0_ represents the level of the intrinsic genetic diversity. Selection on a genomic locus reduces the genetic diversity at the locus. Hitchhiking propagates the effect of selection on the surrounding region. We described the selective effect ($sh_{i}$) on a gene in terms of the long-term molecular evolutionary rate ($r_{i}$) as $sh_{i}=exp\left(\alpha +\beta r_{i}\right)$ (see Materials and Methods). Parameter α describes the impact of recombination relative to long-term rate in the model. Higher α values in genomic regions indicate that recombination contributed more to the prediction of genetic diversity than the regions with lower α values. β describes the impact of the long-term rates of nearby genes. Higher β values in genomic regions indicate that the long-term rates of genes contributed more to genetic diversity than the regions with lower β values. The three parameters were estimated for each chromosome and for each feature (3′-UTR, 5′-UTR, exon, or intron). In total, we obtained 88 (22 chromosomes × 4 features) estimates each for π_0_, α, and β. (**a**–**c**). Pie charts for π_0_, α, and β, respectively, representing the contributions of chromosome, feature, and residuals. The variances of π_0_, α, and β were decomposed into among-chromosome variances, among-feature variances, and variances of residuals in two-way ANOVA that represented the interactions of each set of factors. (**d**–**f**). The among-chromosome variations of π_0_, α, and β, respectively, for each feature (3′-UTR, 5′-UTR, exon, or intron). (**g**). Hierarchical clustering of π_0_, α, and β for each chromosome and feature. We normalized the values of π_0_, α, and β to analyze the three parameters together.

The estimated parameters, π_0_, α, and β, varied among chromosomes (chromosomes 1–22) and features (3′-UTRs, 5′-UTRs, exons, and introns) (Figure 1). π_0_ varied both among chromosomes and features (two-way ANOVA, *p* < 2 × 10^−16^ for chromosomes, *p* < 2 × 10^−16^ for features). However, only features were significant factors for the variations of α (*p* = 0.147 for chromosomes, *p* < 2 × 10^−16^ for features) and β values (*p* = 0.12 for chromosomes, *p* < 2 × 10^−16^ for features) (Figure 1a–c). The estimated mean π_0_ across all chromosomes for 3′-UTR, 5′-UTR, exon, and intron regions were 0.00122, 0.00116, 0.00097, and 0.00110, respectively. Exon regions had the lowest π_0_, followed by intron regions, whereas 3′-UTRs had the highest π_0_ among the four features (Figure 1d). We found that the α and β values were negatively correlated with each other (cor = −0.920, *p* < 2 × 10^−16^). The parameter patterns of 5′-UTRs and exons were similar to each other, whereas those of 3′-UTRs and introns were similar to each other (Figure 1e–f). Hierarchical clustering using fitted values of π_0_, α, and β of all 22 chromosomes showed that 5′-UTRs and exons clustered together, whereas 3′-UTRs and introns clustered together (Figure 1g). These results indicate that the main effect differs for each genetic region. The 5′-UTR and exon regions had lower α and higher β values compared with the 3′-UTR and intron regions. This result indicates that long-term rate is the main effect that impacts genetic diversity at 5′-UTRs and exons, whereas recombination is the main effect that impacts genetic diversity at 3′-UTRs and introns. Thus, we focused on the relationship between long-term rates and gene diversity in this study.

### 3.2. Bridging Micro- and Macroevolution at the Molecular Level

**Figure 2:**
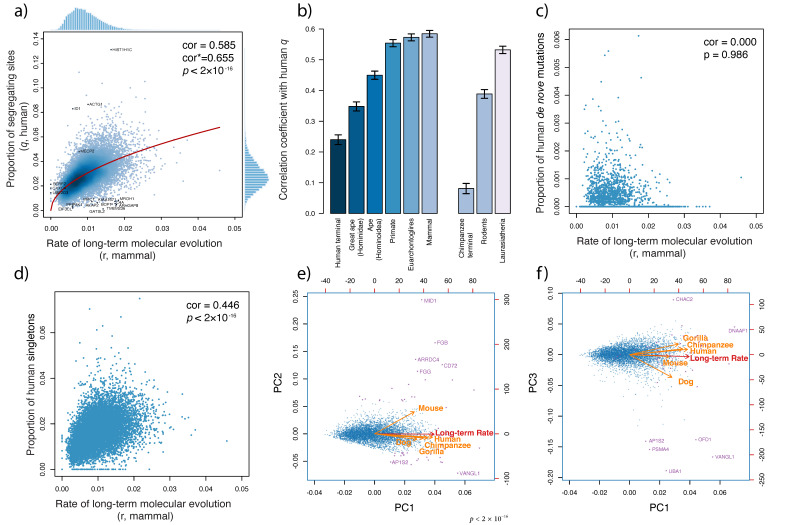
**Bridging micro- and macroevolution at the molecular level:** (**a**). Correlation between the human proportion of segregating sites (q) and the rate of mammalian molecular evolution (r) of 14,671 genes. cor* indicates the bias-corrected correlation. (**b**). Correlation between the human proportion of segregating sites (q) and the rate of molecular evolution (r) based on gene trees of different taxonomic clades. (**c**). Correlation between the proportion of human de novo mutations and the rate of mammalian molecular evolution (r). (**d**). Correlation between the proportion of human singletons and the rate of mammalian molecular evolution (r). (**e**,**f**). Principal component analysis (PCA) of q of five species and the long-term rate using 5560 single-copy genes.

Strong linear relationships were found between mammalian molecular evolutionary rates and human q, the proportion of segregating sites (Figure 2a, cor = 0.585, *p* < 2 × 10^−16^). The bias-corrected correlation was as high as 0.665 (Figure 2a, *p* < 2 × 10^−16^), which indicates that the intraspecific genetic diversity of a gene is thus largely influenced by the molecular evolutionary rate of the gene. Variation in functional constraints on genes is the likely source of this causal relationship [39]. The estimated shape parameter in the negative binomial distribution in humans was as large as 125,785, which indicates that over-dispersion of human q can be ignored, and human q largely followed a Poisson distribution. The correlation between q of four tested non-human mammal species (chimpanzees, orangutans, mice, and dogs/wolves) and their molecular evolutionary rates, which ranged from 0.368 (mice) to 0.478 (chimpanzees), was consistent with the findings in humans (Figure S1, all were significant with *p* < 2 × 10^−16^).

Figure 2b shows the raw correlation between the human proportion of segregating sites (q) and molecular evolutionary rate variation inferred from different taxonomic hierarchies, ranging from the human/chimpanzee divergence (human terminal branches) to family Hominidae (great apes) to superorder Euarchontoglires. Significant correlations between human q and rate variations were found for all clades, with higher taxonomic hierarchies showing stronger correlations. Furthermore, among-gene rate variation in rodents and Laurasiatheria was highly correlated with human q (cor = 0.389 and 0.532, respectively; both *p* < 2 × 10^−16^). The rate variation in mammals had the highest raw correlation with human q (Figure 2b, cor = 0.585). Evolution is a stochastic process, which is affected by multiple factors. A deeper evolutionary scale results in more stable estimates of the long-term rates across genes.

### 3.3. The Immediate Effect of Natural Selection

Among-gene variation of intraspecific genetic diversity is caused by among-gene variation in mutation rate and functional constraints. To examine whether the mammalian molecular evolutionary rate of a gene correlates with its mutation rate, we regressed the rate of point mutations per generation (proportion of human de novo mutations) on mammalian molecular evolutionary rates. We found no correlation between the proportion of de novo mutations and mammalian molecular evolutionary rates (cor = 0.00, *p* = 0.986, Figure 2c). In contrast, the proportion of singletons in the 1000 Genomes Project human data was found to be affected by mammalian molecular evolutionary rates (cor = 0.446, *p* < 2 × 10^−16^; Figure 2c). By applying negative binomial regression to singletons, the expected number of singletons was estimated as:

$$E\left[\mathrm{number}\;\mathrm{of}\;\mathrm{singletons}\right]/\left(\mathrm{gene}\;\mathrm{length}\right)=0.113\times {\left(\mathrm{long}\;-\;\mathrm{term}\;\mathrm{rates}\right)}^{0.413}.$$

The effect of mammalian molecular evolutionary rate was highly significant (*p* < 2 × 10^−16^, Table 1).

Singletons, which reflect relatively recent changes, may help shape complex traits [40,41]. Selection caused by long-term evolutionary force has in turn influenced human genetic diversity over a short time scale, probably soon after the occurrence of the initial mutation.

### 3.4. Commonality and Specificity among Species

The principal component analysis of the genetic diversity and the long-term molecular evolutionary rate showed the commonality and specificity of genetic diversity among five species, human, chimpanzee, gorilla, mouse, and dog/wolf (Figure 2e–f). The first principal component (PC1) explained 48% of variability and expressed the overall amount of inter- and intraspecific genetic diversity. PC2 had a contribution of 13.3% and represented reduced/enhanced functional constraints in mice. PC3 had a contribution of 13.0% and expressed enhanced/reduced functional constraints in dogs/wolves. The relationships among the five species precisely reflected their phylogenetic relationships.

The top 20 genes with negative deviations in PC1 are generally those genes that have enhanced functional constraints in mammals. These genes were enriched in the categories of learning or memory (GO:0007611, FDR = 0.0035) and behavior (GO:0007610, FDR = 0.004) (Table S1) in a Biological Process (GO) enrichment search [42]. The top 20 genes in the positive direction of PC2, which had relaxed functional constraints or were subjected to diversifying selection in mice, were enriched in fibrinogen complex (GO:0005577, FDR = 1.87 × 10^−5^, Table S1). The MID1 gene, which had the largest deviation in PC2, was previously reported to have undergone X-inactivation in humans, but escaped in mice [43]. For the top 20 genes in PC3, a GO enrichment search did not suggest any significant categories. However, a Reactome Pathways search [44] revealed that these genes are mostly enriched in Class I MHC-mediated antigen processing and presentation and the adaptive immune system (seven genes, Table S1: AP1S2, CDC27, PSMA4, PSMC2, TLR4, UBA1, and UBE2L6). The adaptive immune system was subjected to enhanced functional constraints in dogs/wolves, probably because the carnivorous lifestyle of their wild ancestors was accompanied by a higher risk of pathogen infections.

### 3.5. Enhanced/Reduced Functional Constraints on the Genes

**Figure 3:**
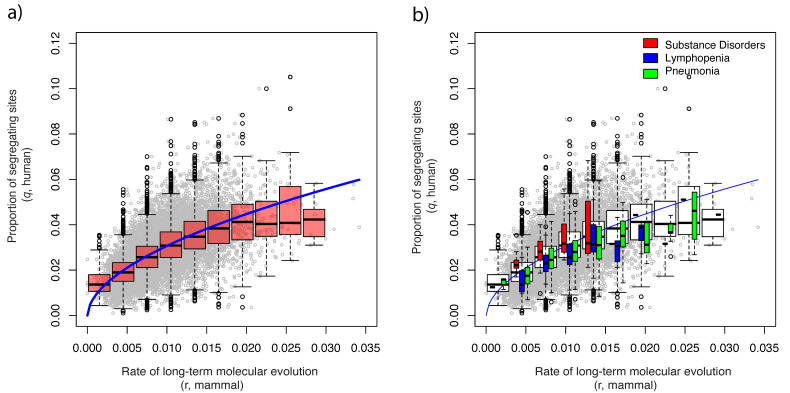
**Disease-associated gene set test:** (**a**). Scatter plot, boxplot, and regression model of the human proportion of segregating sites (q) vs. mammalian molecular evolutionary rates of 14,671 genes. The blue curve represents the prediction by the negative binomial regression (see Methods). (**b**). Examples of significant disease-associated gene sets in humans revealed by analysis of 14,267 disease-associated gene sets. Genes related to the following diseases in humans are shown as examples: substance-related disorders, lymphopenia, and pneumonia.

Species-specific enhanced/reduced functional constraints of each gene can be quantified by measuring the deviation in the proportion of segregating sites (q) from predictions based on mammalian molecular evolutionary rates. Figure 3 shows how such deviation can be used to predict species-specific selections on gene (Figure 3a) and disease-related gene sets (Figure 3b), respectively. Genes or gene-sets with positive deviations indicate they are under relaxed functional constraints or under positively selected, while genes or gene sets with negative deviations indicate they are under enhanced functional constraints. In Figure 3b, gene sets associated with substance-related disorders, lymphopenia, and pneumonia are used as examples. It indicates that the functional constraints on the genes associated with substance-related disorders were reduced in human, whereas those associated with lymphopenia and pneumonia were enhanced. Table S2 summarizes the *p*-values and *t*-values of all 14,267 disease-related gene sets.

#### 3.5.1. Genes under Species-Specific Selection

**Figure 4:**
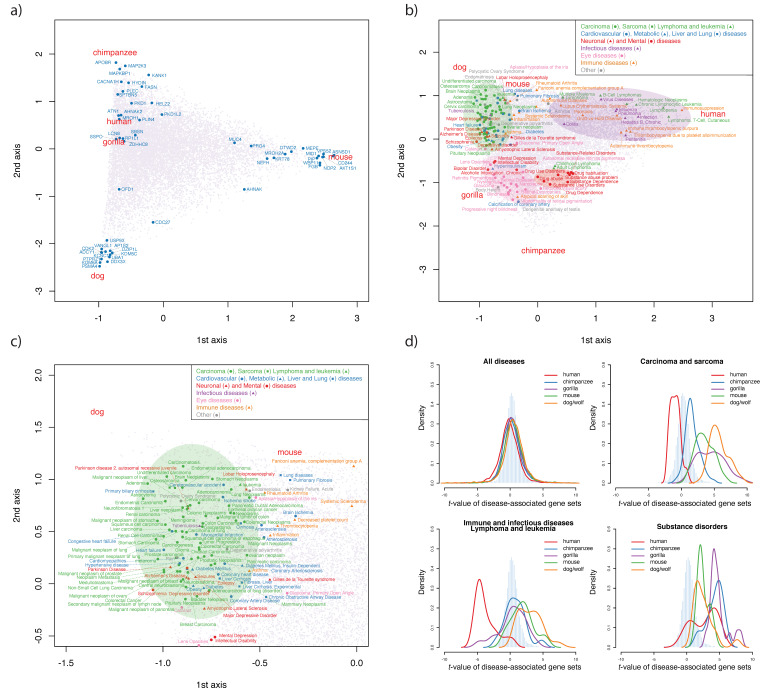
**Correspondence analysis of genes and disease-associated gene sets:** (**a**). Correspondence analysis of 14,671 genes of five species using $-\log_{10}\left(p\right)$ of each gene. (**b**,**c**). Correspondence analysis of 14,267 disease-associated gene sets of five species using $-\log_{10}\left(p\right)$ of each disease-associated gene set. The diseases highlighted are significant diseases with a false discovery rate of 0.01. (**d**). Distribution of *t*-values of all diseases and significant disease-associated gene sets detected by correspondence analysis. The pale blue histogram indicates the distribution of average *t*-values of five species for each disease.

Figure 4a shows the correspondence analysis of genes and species based on the deviance the genetic diversity from the predicted values from the long-term molecular evolutionary rate (see Method). With FDR set to 0.01, we detected 53 genes whose diversity can explain the species-specific diversity, among which 24 were designated as being related to disease by the UniProt database [45] (Table S3). Based on their locations, we clustered the genes into three groups: apes, mice, and dogs/wolves. The mouse group was enriched in structural molecule activity (GO: 0005198, FDR = 0.003), whereas the other two groups did not have any significant enrichment. Notably, the genes detected by PCA and CA largely overlapped, especially those that were most important for different PCs and groups (e.g., MID1 and FGB for mice, and UBA1 and VANGL1 for dogs/wolves; Figure 2e–f and Figure 4a).

#### 3.5.2. Multiple Species Disease-Associated Gene Set Test

With FDR of 0.01, we identified 20 disease-associated gene sets for humans, 23 for chimpanzees, 86 for gorillas, 31 for mice, and 130 for dogs/wolves (Table S4). To capture the patterns of species-specific evolution on the traits revealed by disease-associated genes, we conducted CA based on the negative log-transformed *p*-values (Figure 4b–c). With an FDR of 0.01, 180 gene sets were significant for at least one species (Table S5). Based on the disease category annotations in DisGenNET (V6.0) [29] and the MalaCard database [30], these were assigned to six major categories, including cancers (such as carcinoma, sarcoma, lymphoma, and leukemia), genetic diseases (such as cardiovascular, metabolic, and liver and lung diseases), neuronal and mental diseases, infectious diseases, eye diseases, and immune diseases. We marked particular clustering of disease-associated gene sets for each species, and defined four groups: “cancer and organ diseases”, “immune and infectious diseases”, “substance disorders”, and “eye diseases”. Figure 4d shows the distributions of *t*-values of major diseases in each group. Notably, the significant diseases detected for each species (Table S4) and CA (Table S5) were also largely consistent.

#### 3.5.3. Cancer Disease Gene Sets and Morphological Evolution

The prominent diseases detected for mice and dogs/wolves were largely cancer diseases with positive *t*-value (Figure 4d; Table S4). The “cancer and organ diseases” group was also located near mice and dogs/wolves in CA (Figure 4b–c). These results indicate that organ development was the most significant character that differentiated mice and dogs/wolves from great apes.

Carcinomas are epithelial cell-derived cancers, whereas sarcomas are connective tissue-derived cancers. Our testing datasets included both types of cancers, but the prominent cancers detected for each species by CA was largely carcinoma of different organs. Only one sarcoma (osteosarcoma) was detected. The evolution of epithelial cells rather than connective tissue may thus be the main contributor to the morphological evolution of different species.

Interestingly, great apes, especially humans, have relatively low *t*-values of cancer diseases compared with mice and dogs/wolves (Figure 4d). Compared with those species, cancer-related genes in great apes are subjected to enhanced functional constraints. Humans have evolved much longer lifespans (122.5 years by [46]) than other great apes (e.g., chimpanzee, 68 years) and dogs (27 years), while mice live for less than 4 years [46,47]. Cancer-associated gene sets that are subjected to enhanced functional constraints in humans may be related to the evolutionary acquisition of longer lifespan.

#### 3.5.4. Echoes of Infectious Diseases in the Human Genome

Outliers in humans were largely infectious diseases, immune diseases, lymphoma, and leukemia, which had negative *t*-values (FDR = 0.01). The “immune and infectious disease” group was also located near humans in CA (Figure 4b). Influenza-related gene sets had the highest significance in humans (*t*-value = −5.62), and pneumonia (*t*-value = −5.17) and chronic hepatitis B (*t*-value = −4.51) were also highly significant. With FDR as 0.1, more infectious disease-related gene sets were detected in humans, such as Saint Louis encephalitis, adult T-cell lymphoma/leukemia, hepatitis C, dengue fever, and brucellosis. Several of these diseases have caused multiple historically important pandemics and taken lives (e.g., Saint Louis encephalitis [48] and dengue fever [49]); although, some of them still cause severe public health problems (e.g., influenza and pneumonia [50]). These diseases affected human populations worldwide, leading to a strong selective sweep, which is consistent with the finding in previous study [51].

#### 3.5.5. Social Structure and Natural Reward Pathway in Great Apes

Humans, chimpanzees, and gorillas shared several outliers of substance-related disorders, and they were all highly significant (Table S4). In CA, a related group was also located in the middle of three great apes. The positive *t*-values associated with substance-related disorders indicate that their specificity among mammals is due to evolution of the natural reward pathway in the motivational–emotional system.

We also note that there were several gene sets in the vicinity of great apes associated with diseases affecting retina development and eye structures. This may be related to the evolution of color vision in primates: in eutherian mammals, only primates have true trichromatic color vision [52].

## 4. Discussion

The genetic diversity of human genomes gradually accumulated during tens of thousands of years of evolution, and was affected by many factors such as selection, mutation, and migration [1,2,3]. Purifying selection due to functional constraints predominantly removes deleterious variations in coding regions and functional non-coding regions such as tRNA, rRNA, or long functional non-coding RNAs [4,6,53]. Other genetic regions are possibly neutral or were impacted by selective sweeps or background selection [4,5]. Pouyet et al. 2018 found that as much as 85% of the human genome was under background selection [15]. This result challenges the general idea that population genomics can accurately estimate population history. Because the existing procedures of population history inference mostly assume the neutrality of mutations, the target polymorphic sites for inference need to be cautiously sampled from the genomic regions with high recombination rates larger than 1.5 cM/Mb.

Because nearly whole genomic regions are under background selection, characterizing the selection is the first step toward understanding genomic diversity. We found that the long-term molecular evolutionary rates of genes are highly correlated with their intraspecific genetic diversity. Growing genomic data of an increasing number of species enables accurate inference of phylogenetic gene trees; this provides a reason to use long-term evolutionary rates as predictors of intraspecific genetic diversity. We tested the effect of long-term evolutionary rates and recombination on shaping genetic diversity in both coding regions (exons) and non-coding regions (3′-UTRs, 5′-UTRs, and introns), and found that the long-term rate is the main force shaping genetic diversity in 5′-UTRs and exons. Further analysis showed that a large quantity of de novo mutations does not contribute to intraspecific genetic diversity. Once the baseline of species-level genetic diversity can be predicted by mammalian long-term rate, the enhanced/reduced functional constraints of each gene can be quantified by measuring the deviation in species-level genetic diversity from predictions based on the mammalian long-term rates. We estimated human species-specific evolution by both gene and disease gene sets.

The genes related to infectious diseases and substance-related disorders were the most representative examples of adaptive evolution in human genome. Genes related to infectious diseases were under strong purifying selection, possibly because the modern society of human populations increased the chance of their exposure to pathogens. Substance-related disorders were under reduced functional constraints. They represent a reliance and/or abuse of a certain substance. The mechanism underlying these disorders stems from the effect of the “substance” (e.g., a drug) on the natural reward pathway of the motivational–emotional system. In nature, reward pathways generate positive emotions that motivate organisms to search for food, water, mates and other beneficial environmental resources, and to avoid predators [54]. Great apes display much more complex social behavior compared with all other mammals; in particular, humans have the most complex societal culture of all living organisms and enjoy the largest array of “natural rewards,” including science, art, literature, and music. Motivation is a basic driver of human behavior, and positive emotion is at its core [55]. We tested 14,267 disease-related gene sets, and reported whether these gene sets are under relaxed or strengthened functional constraints. These results offer new insights into human species-specific adaptations.

In this paper, we showed the effect of the long-term evolutionary rates of genes on the genetic diversity of coding regions and their surrounding regions. However, a preliminary analysis indicated that the adopted model did not explain the variable genetic diversity well in intergenic regions that were more than 100,000 bp away from the coding regions. This suggests that there remain some unknown influential factors that shape the variability of genetic diversity in intergenic regions. There are approximately 500 ultraconserved elements in the human genome, the majority of which are in the intergenic regions and function as enhancers that activate tissue-specific gene expression during embryonic development. They are depleted at topologically associating domain boundaries and enriched inside domains. Comparative analysis of intra- and interspecific genetic diversity in the intergenic regions will help bridge the great divide between the two extremes, and clarify the role of ultraconserved regions and regions that are free from background selection.

## Figures and Tables

**Table 1:** Negative binomial regression of numbers of human de-novo mutations and singletons on the median branch length of the mammalian gene trees. The regression model is log(E[counts]/(gene length))=α+βlog(median branch length): $E\left[\mathrm{counts}\right]/\left(\mathrm{gene}\;\mathrm{length}\right)=e^{\alpha }{\left(\mathrm{long}\;-\;\mathrm{term}\;\mathrm{rates}\right)}^{\beta }$.

	de novo Mutation			Singleton		
	Estimate	SE	*p*-Value	Estimate	SE	*p*-Value
intercept	−9.938	0.307	<2 × 10^−16^	−2.177	0.026	<2 × 10^−16^
log(Long-term rates)	−0.008	0.064	0.904	0.413	0.006	<2 × 10^−16^

## Data Availability

Source data and gene trees are available at https://github.com/wujiaqi06/Long-Term-Rate.

## Supplementary Materials

The following supporting information can be downloaded at: https://www.mdpi.com/article/10.3390/genes13040708/s1, Figure S1. Correlations between the proportion of segregating sites (q) and the rates of mammalian molecular evolution (r) of chimpanzees, gorillas, mice, and dogs/wolves. The result was obtained from 12,236 genes that are shared by all five species; Table S1: Top 20 genes for the three principal components (PC; PC1–PC3) in principal component analysis. The gene enrichment results are also summarized in this table; Table S2: *p*-values and *t*-values of each disease from the disease-associated gene set tests of all 14,267 diseases of humans, chimpanzees, gorillas, mice, and dogs/wolves; Table S3: Significant genes detected by correspondence analysis with a false discovery rate of 0.01. The gene enrichment results are also summarized in this table; Table S4: Significant disease-associated gene sets in humans, chimpanzees, gorillas, mice, and dogs/wolves, with a false discovery rate of 0.01; Table S5: Significant disease-associated gene sets detected by correspondence analysis using $-\log_{10}\left(p\right)$ of each disease-associated gene set, with a false discovery rate of 0.01.
