# Supplementary figures and images for: Molecular Evolutionary Rate Predicts Intraspecific Genetic Polymorphism and Species-Specific Selection
Source: Genes (Basel). 2022 Apr 17;13(4):708. doi: 10.3390/genes13040708 (PMC9031814; doi:10.3390/genes13040708)

**Chimpanzee**

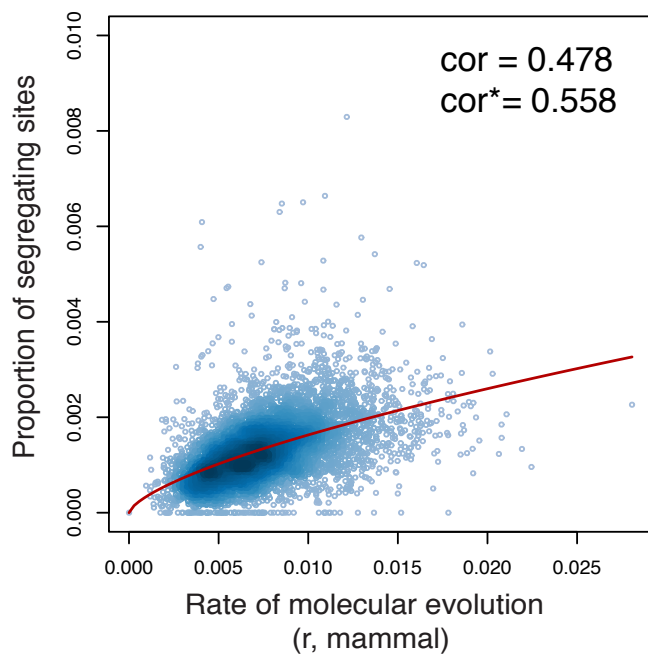

**Gorilla**

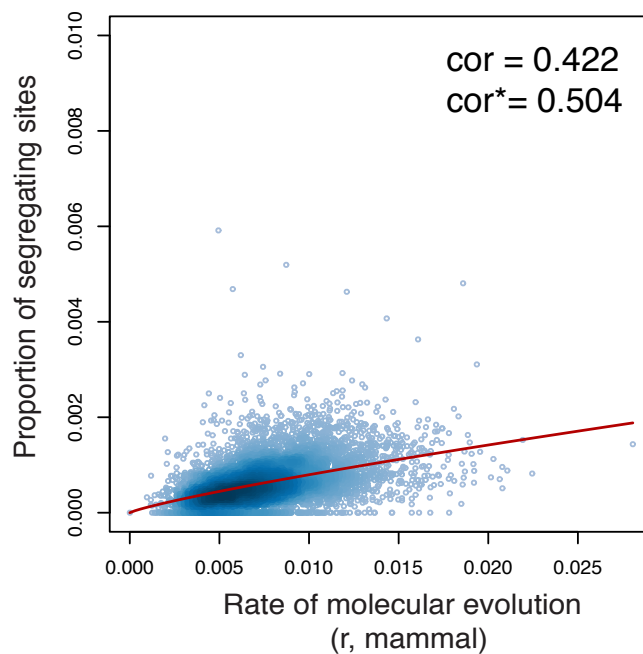

**Mouse**

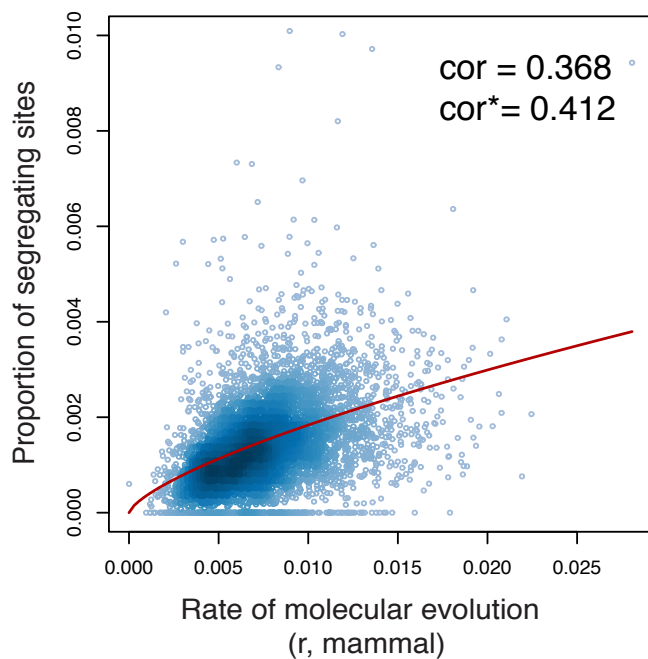

**Dog/wolf**

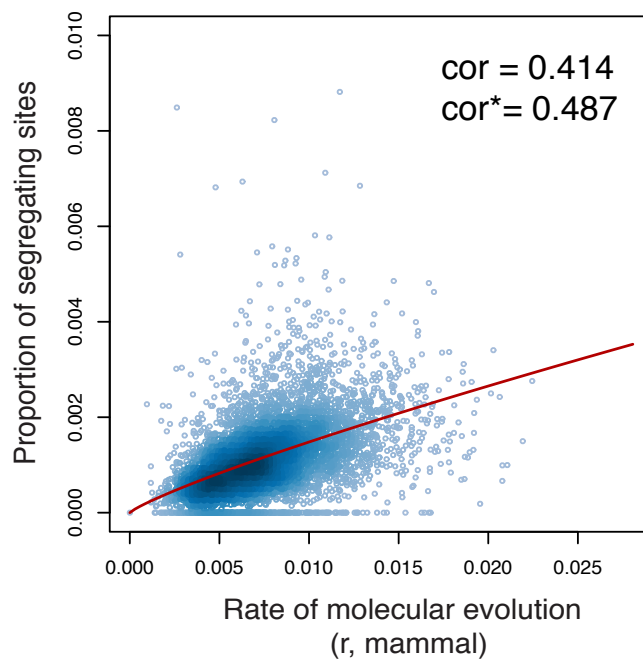

Supplement: Supplementary file 1 [file genes-13-00708-s001.zip › SupplimentaryMaterials/FigureS1.pdf]
